# Supplementary material for: WordCommentsAnalyzer: A windows software tool for qualitative research
Source: F1000Res. 2018 Sep 4;7:536. Originally published 2018 May 3. [Version 2] doi: 10.12688/f1000research.14819.2 (PMC6137411; doi:10.12688/f1000research.14819.2)
Supplement: Supplementary file 3 [file f1000research-7-17614-s0002.tgz › b2a79496-4ada-47c4-9682-a99ce867836d.zip!/Eight Iranian Health Journals/Archives of Rehabilitation_42.docx]

© Archives of Rehabilitation. This work is licensed under CC BY-NC 4.0 (https://creativecommons.org/licenses/by-nc/4.0/).

Volume & Issue: دوره 10 - شماره 2

Title: بررسی توزیع فشار کف پا در افراد بزرگسال سالم هنگام ایستادن و راه‌رفتن

http://rehabilitationj.uswr.ac.ir/article-1-325-fa.html

هدف: مطالعه حاضر به منظور بررسی توزیع فشار کف پا در افراد بزرگسال سالم انجام گردید تا بر اساس آن معیار مناسبی در زمینه توزیع طبیعی فشار کف پا معرفی شود. روش بررسی: در این مطالعه توصیفی- تحلیلی،80 مرد و زن با پای سالم از نمونه‌های در دسترس به صورت ساده انتخاب و با استفاده از دستگاه اندازه‌گیری فشار RS-Scan فشار کف پا در نه منطقه آناتومیکی هنگام ایستادن و راه‌رفتن اندازه‌گیری شد. داده‌های حاصل با استفاده از آزمون‌های آماری تی‌زوجی و تی‌مستقل مورد تجزیه و تحلیل قرار گرفتند. یافته‌‌ها: مناطق پاشنه با 31/43 ، سرهای استخوان‌های کف‌پایی دوم و سوم با 30/86 و 28 و انگشت شست با 23/51 نیوتن بر سانتی‌متر مربع دارای بیشترین فشار حین راه‌رفتن بوده و کمترین فشار در انگشت دو تا پنج و ناحیه میانی پا قرار داشت. میزان فشار استاتیک در ناحیه جلوی پای چپ (P<0/001)، درصد تماس در ناحیه عقب هر دو پا (P<0/001) و ناحیه میانی پای راست (P=0/02) و مقدار حداکثر فشار طی راه‌رفتن در نواحی استخوان‌های کف‌پایی چهارم پای راست (۰/۰۵=P) و چپ (۰/۰۴=P) و پنجم پای راست (۰/۰۲=P) و پاشنه هر دو پا (۰/۰۰۱>P) بین مردان و زنان تفاوت معناداری داشت. نتیجه‌گیری: تفاوت‌های زیادی بین نحوه توزیع و میزان فشار کف‌پای زنان و مردان وجود دارد که باید در موارد مختلف از جمله طراحی کفش، روندهای درمانی و توانبخشی و همچنین پاهای در معرض خطر مانند افراد دیابتی مورد توجه قرار گیرد.

Title: مقایسه الگوی فعالیت عضله راست‌کننده ستون‌فقرات در حین عمل پایین گذاشتن بار قبل و بعد از حفظ طولانی مدت وضعیت فلکسیون کمر در افراد با سابقه کمردرد و افراد سالم

http://rehabilitationj.uswr.ac.ir/article-1-326-fa.html

هدف: مقایسه پدیده فلکسیون ریلکساسیون در عضله راست‌کننده ستون‌فقرات در حین عمل پایین‌گذاشتن بار قبل و بعد از حفظ طولانی مدت وضعیت فلکسیون کمر در افراد با سابقه کمردرد و افراد سالم هدف این مطالعه است. روش بررسی: در این مطالعه تجربی و مداخله ای، 15 فرد با سابقه کمردرد با نمونه‌گیری ساده و در دسترس و 15 فرد سالم با روش جورکردن با بیماران انتخاب و فعالیت عضله راست‌کننده ستون‌فقرات آنها توسط الکترومیوگرافی سطحی و میزان فلکسیون تنه توسط الکتروگونیومتر اندازه‌‌گیری و ثبت شد. افراد مورد مطالعه عمل باربرداشتن و پایین گذاشتن بار را در شرایط بدون وزنه و با وزنه انجام می‌دادند. سپس حفظ طولانی مدت وضعیت فلکسیون کمر به مدت 7 دقیقه انجام شده و مجدداً عمل باربرداشتن و پایین‌گذاشتن بار تکرار می‌شد. قطع فعالیت عضله راست‌کننده ستون‌فقرات در حین عمل پایین گذاشتن بار یا پدیده فلکسیون ریلکساسیون قبل و بعد از حفظ طولانی مدت وضعیت فلکسیون کمر در شرایط بدون وزنه و با وزنه در دو گروه مقایسه و با استفاده از آزمون آماری تحلیل واریانس سه طرفه مختلط برای بررسی اثر متقابل وضعیت در بار در گروه مورد تجزیه وتحلیل قرار گرفت. یافته‌ها: مطابق نتایج حاصل اثر متقابل وضعیت در بار (P=0/120) و گروه در بار (۰/۳۶۵=P) معنادار نبود، ولی اثر گروه در وضعیت معنادار بود (۰/۰۰۱>P) ، به‌گونه‌ای که تأثیر حفظ طولانی مدت وضعیت فلکسیون کمر در گروه با سابقه کمردرد معنادار بود (۰/۰۰۱>P)، ولی در افراد سالم معنادار نبود (۰/۴۶۸=P). در افراد با سابقه کمردرد پدیده فلکسیون ریلکساسیون بعد از حفظ طولانی مدت وضعیت فلکسیون کمر، نسبت به قبل از آن دیرتر رخ می‌داد. نتیجه‌گیری: در افراد با سابقه کمردرد، پدیده فلکسیون ریلکساسیون بعد از حفظ طولانی مدت وضعیت فلکسیون کمر، دیرتر از قبل از حفظ وضعیت مذکور اتفاق می‌افتد، در حالی‌که در افراد سالم تفاوتی بین قبل و بعد از حفظ طولانی مدت وضعیت فلکسیون کمر نیست.

Title: تأثیر آموزش خودمراقبتی بر کیفیت‌زندگی بیماران مبتلا به نارسایی‌قلبی

http://rehabilitationj.uswr.ac.ir/article-1-327-fa.html

هدف: نارسایی‌قلبی یکی از شایع ترین اختلالات قلبی- عروقی بوده و به عنوان یک اختلال مزمن، پیشرونده و ناتوان کننده مطرح می‌باشد. کیفیت‌زندگی در بیماران مبتلا به نارسایی‌قلبی پائین است. یکی از روش‌های ارتقا‌دهنده کیفیت‌زندگی، آموزش خودمراقبتی می‌باشد. هدف مطالعه حاضر تعیین تأثیر آموزش خودمراقبتی بر کیفیت‌زندگی بیماران مبتلا به نارسایی‌قلبی می‌باشد. روش بررسی: در این مطالعه نیمه تجربی 126 بیمار مبتلا به نارسایی‌قلبی از بین مراجعه کنندگان به بیمارستان قلب و عروق شهید رجایی تهران مطابق معیارهای تحقیق انتخاب و با روش بلوک‌های تبدیل شده تصادفی به طور مساوی در دوگروه مداخله و کنترل قرار گرفتند. ابزار گرد آوری داده‌ها شامل فرم مشخصات دموگرافیک، پرسشنامه کیفیت‌زندگی بیماران مبتلا به نارسایی‌قلب و "پرسشنامه اروپایی رفتار مراقبت از خود بیماران نارسایی‌قلبی" بود. بیماران گروه مداخله در شش گروه ده نفری جهت آموزش قرار گرفته و در 4 تا ۶ جلسه آموزش دیدند و در نهایت بسته آموزشی در اختیار این بیماران قرار گرفت. طول مدت جلسات 30 تا ۴۵ دقیقه بود. برای تحلیل داده‌ها از آزمون‌های تی‌زوجی و مستقل، کای اسکوئر ،کلموگروف- اسمیرنوف، لیون و آنالیز کوواریانس استفاده شد. یافته‌ها: میانگین کیفیت‌زندگی، (P=0/214) و خودمراقبتی (P=0/795) بین گروه مداخله و کنترل قبل از انجام مداخله تفاوت معنادار نداشت، ولی بعد از مداخله تفاوت دو گروه در این ۲ متغیر معنادار شد (P<0/001). میانگین ابعاد اقتصادی-اجتماعی، روانی، جسمی- فعالیت و سلامت عمومی کیفیت‌زندگی بین دو گروه بعد از انجام مداخله تفاوت معنادار داشت (P<0/001). کیفیت‌زندگی (P=0/154) و رفتارهای مراقبت از خود (P=0/431) در گروه کنترل قبل و بعد از مداخله تفاوت معنادار نداشته، ولی در گروه مداخله این متغیرها بعد از مداخله نسبت به قبل از آن تفاوت معناداری یافته بود (P<0/001). نتیجه‌گیری: نتایج مطالعه نشان داد که آموزش خودمراقبتی بر کیفیت‌زندگی بیماران مبتلا به نارسایی‌قلبی مؤثر می‌باشد و توصیه می‌شود به عنوان یکی از روش‌های اثربخش و غیر دارویی، جهت ارتقاء کیفیت‌زندگی این بیماران به‌کار برده شود.

Title: تأثیر برنامه بازتوانی از طریق پیاده‌روی منظم بر وضعیت‌ بالینی افراد مبتلا به بیماری‌های انسدادی مزمن ریه

http://rehabilitationj.uswr.ac.ir/article-1-328-fa.html

هدف: بیماری انسدادی مزمن ریه بیماری است که با محدودیت جریان هوا در ریه مشخص می‌شود، به‌طوری‌که این محدودیت به‌طور کامل قابل برگشت نیست. هدف از این پژوهش تعیین تأثیر پیاده‌روی برنامه ریزی شده به عنوان برنامه بازتوانی بر وضعیت‌بالینی (نتایج اسپیرومتری، خلط، تنگی نفس و...) بیماران انسداد مزمن ریه با شدت متوسط می‌باشد. روش بررسی: در این پژوهش نیمه تجربی از بیمارستان‌های آموزشی منتخب شهر اصفهان 100 نفر از بیماران انسداد مزمن ریه با شدت متوسط به روش نمونه‌گیری در دسترس انتخاب و نهایتاً 30 نفر در گروه شاهد و 50 نفر در گروه آزمون باقی‌ماندند. ابزار گردآوری داده‌ها، پرسشنامه اطلاعات دموگرافیک و برگ ثبت علائم و نشانه‌های وضعیت‌بالینی است. روایی آن ازطریق روایی محتوی و پایایی آن ازطریق پایایی همزمان سنجیده شد و اطلاعات قبل از پژوهش، 4 هفته بعد و 2 ماه بعد از مداخله در هر دو گروه جمع آوری شد. مداخله در گروه آزمون به‌صورت دو جلسه کلاس آموزشی در مورد ماهیت بیماری و اثرات ورزش (پیاده‌روی) بر روی وضعیت سلامت برگزار شد و پیاده‌روی برنامه‌ریزی شده سه روز در هفته به میزان 10 تا 35 دقیقه در بیمارستان با راهنمایی و همراهی پژوهشگر به مدت 4 هفته انجام و سپس ادامه برنامه پیاده‌روی بیماران در منزل دنبال و در برگه ثبت انجام پیاده‌روی گزارش شد. پی‌گیری به مدت 2 ماه صورت گرفته و سپس نتایج برگه ثبت وضعیت‌بالینی و اسپیرومتری دو گروه با استفاده از آزمون‌های آماری تحلیل مکرر با تست ویلکز، تی‌مستقل و تی‌زوجی مورد تجزیه و تحلیل و مقایسه قرار گرفت. یافته‌ها: میانگین نمره تنگی نفس (۰/۱=P)، کیفیت خلط (۰/۴۵=P)، الگوی تنفسی (۰/۰۶=P) و صداهای ریوی (۰/۰۶=P) قبل از شروع، 4 هفته بعد و 2 ماه بعد مطابق آزمون تحلیل مکرر تفاوت معناداری در گروه شاهد نشان نداد، ولی در گروه آزمون تفاوت معنادار بود (مقادیر احتمال به ترتیب ۰/۰۱، ۰/۰۰۱>، ۰/۰۰۴ و ۰/۰۳). همچنین تست عملکرد ریوی (اسپیرومتری) در ماه دوم بین گروه‌های کنترل و آزمون براساس آزمون تی‌مستقل تفاوت معناداری داشت (0/001>P). نتیجه‌گیری: برنامه پیاده‌روی منظم به عنوان برنامه بازتوانی در بیماران انسداد مزمن ریه باعث بهبود وضعیت‌بالینی (کاهش سرفه و میزان خلط و الگوی تنفسی و صداهای ریوی منظم و طبیعی) و عملکرد ریوی مناسب می‌شود و پیشنهاد می‌شود پیاده‌روی برنامه‌ریزی شده براساس زمان 10 تا 35 دقیقه و 3 بار در هفته به عنوان برنامه بازتوانی در این بیماران پیگیری شود.

Title: تأیید روایی سازه نسخه فارسی مجموعه آزمون ارزیابی شناختی کاردرمانی لونستین (لوتکا)

http://rehabilitationj.uswr.ac.ir/article-1-329-fa.html

هدف: اسکیزوفرنیا عموماً توأم با نقص شناختی است. به دلیل تأثیر نقایص شناختی برتمامی ابعاد زندگی، ارزیابی شناخت با تقویت طرحهای درمانی، به کسب استقلال در زندگی روزمره نیز کمک می نماید. هدف از این پژوهش مقایسه کارکرد شناختی بیماران مبتلا به اختلال اسکیزوفرنیا و افراد عادی جامعه برای تأیید روایی سازه مجموعه ارزیابی شناختی کاردرمانی لونستین (لوتکا) جهت استفاده در ارزیابی‌‌های بالینی بیماران اسکیزوفرنیا است. روش بررسی: این پژوهش از نوع اعتبار سنجی و روا سازی ابزاری است که به‌صورت مقایسه‌ای و بر روی 737 نفر (237 بیمار و 500 افراد عادی ) 20 تا 60 ساله انجام شد. نمونه گیری تصادفی و متناسب با ملاکهای آزمون بود. ابزار مورد نظر آزمون ارزیابی شناختی کاردرمانی لونشتین بود که شش حوزه از شناخت را مورد ارزیابی قرار می دهد. داده‌‌ها از طریق پرسشنامه اطلاعات دموگرافیک و آزمون لوتکا جمع آوری و به کمک آزمون‌های آماری لیون، تی‌مستقل و ضریب همبستگی مورد تجزیه و تحلیل قرار گرفت. یافته‌‌ها: بین وضعیت شناختی بیماران اسکیزوفرنیک و افراد عادی جامعه در تمامی هفت خرده‌آزمون تفاوت معنادار وجود داشت (۰/۰۰۱>P). مقایسه کارکرد شناختی بیماران سرپایی و بستری نیز در زمینه درک زمان و مکان، ادراک بینایی، سازماندهی بینایی حرکتی، عملیات ذهنی، توجه‌و‌تمرکز تفاوت معناداری را نشان داد (۰/۰۰۱>P). همچنین برطبق یافته‌‌ها عملیات ذهنی (۰/۰۳۳=P) و توجه ‌و ‌تمرکز (۰/۰۰۷=P) در خانم‌ها بیشتر از آقایان بود. با افزایش سن نمرات اکتسابی تمامی خرده آرمونهای مورد بررسی کاهش داشت. میانگین نمرات افراد شاغل در هفت خرده‌آزمون به صورت معناداری بالاتر از افراد بیکار بود (۰/۰۰۱>P). میانگین نمرات اکتسابی افراد متأهل در خرده‌آزمون درک زمان و مکان «جهت‌یابی» بالاتر بود (۰/۰۳۳=P) و همچنین میان سطح تحصیلات و مطلوبیت هریک از هفت خرده‌آزمون رابطه معناداری وجود داشت (۰/۰۰۱>P). نتیجه‌گیری: همان‌گونه که پیش‌تر اختلال و نقایص شناختی در افراد اسکیزوفرنیک با روش‌ها و آزمون‌های دیگر ثابت شده است، نتایج تحقیق حاضر که با استفاده از آزمون لوتکا می‌باشد، بر این اختلال صحه می‌گذارد. لذا نسخه فارسی آزمون لوتکا ابزار معتبر و مناسبی برای ارزیابی اختلال در کارکرد شناختی بزرگسالان مبتلا به اسکیزوفرنیا بوده و قادر است حیطه‌‌های اختلال شناختی در آنها را تعیین کند.

Title: بررسی تأثیر تمرینات کلیدی مفصل شانه بر گشتاور ایزومتریک حرکات مفصل گلنوهومرال در زنان جوان سالم

http://rehabilitationj.uswr.ac.ir/article-1-331-fa.html

هدف: این مطالعه به‌منظور بررسی میزان تغییرات گشتاور ایزومتریک برخی از حرکات مفصل گلنوهومرال، بعد از انجام تمرینات کلیدی این مفصل انجام شد. روش بررسی: در این کارآزمایی بالینی تصادفی 36 دختر جوان سالم به‌طور تصادفی انتخاب و با روش تصادفی تعادلی در دو گروه مداخله و کنترل قرار گرفتند. آزمودنی‌ها در گروه مداخله به مدت شش هفته، هفته‌ای سه جلسه، تمرینات کلیدی را طبق روش دلورم (Delorm) به‌صورت وزنه آزاد انجام ‌دادند. قبل و بعد از شش هفته، گشتاور ایزومتریک حرکات مورد بررسی در دو گروه توسط دستگاه دایناتورک در وضعیت‌های آزمون مربوط به هر کدام از حرکات مورد بررسی اندازه‌گیری شد، به‌طوری‌که آزمودنی‌ها در آزمون مربوط به هر حرکت، سه بار حداکثر انقباض ایزومتریک آن حرکت را انجام داده و میانگین این سه بار به عنوان قله گشتاور برای ارزیابی گشتاور مورد نظر محسوب شد. داده‌های حاصل با استفاده از آزمون‌های آماری کولموگروف-اسمیرنوف، تی‌زوجی و تی‌مستقل مورد تجزیه و تحلیل قرار گرفت. یافته‌ها: در گروه مداخله، گشتاور ایزومتریک حرکات مورد بررسی بعد از شش هفته تمرین نسبت به قبل از آن افزایش معناداری یافت (P<0/001)، ولی در گروه کنترل هیچ‌گونه تغییر معناداری در هیچ‌یک از حرکات مشاهده نشد (P>0/05). همچنین مقایسه دو گروه قبل از مداخله در هر شش حرکت مورد بررسی حاکی از عدم تفاوت معنادار بین آنها بود (P>0/05)، ولی بعد از مداخله بین دو گروه در هر شش مورد تفاوت معناداری ایجاد شد (P>0/001). نتیجه‌گیری: تمرینات کلیدی و اختصاصی معرفی‌شده برای مفصل گلنوهومرال، باعث افزایش گشتاور حرکات این مفصل می‌شود؛ بنابراین در برنامه تمرینی افراد سالم به‌ویژه افراد با ورزش‌های بالای سر می‌توان از این تمرینات استفاده نمود.

Title: مقایسه حس‌ عمقی مفصل شانه بین زنان والیبالیست و زنان غیرورزشکار

http://rehabilitationj.uswr.ac.ir/article-1-332-fa.html

هدف: حس‌عمقی در ثبات داینامیک مفصل گلنوهومرال پرتاب‌گران و ورزشکارانی که حرکات ناگهانی و پرتابی اندام فوقانی دارند، با توجه به شل بودن قابل ملاحظه کپسول مفصلی و دامنه حرکتی بیش از حد آنها اهمیت دارد. هدف از این پژوهش مقایسه حس‌عمقی (حس وضعیت مفصل و حس حرکت) بین دو گروه زنان والیبالیست و زنان غیرورزشکار می‌باشد. روش بررسی: به روش نمونه‌گیری ساده و در دسترس و لحاظ‌کردن معیارهای موردنظر 15 والیبالیست زن از تیم‌های دسته اول تهران و 15 زن غیرورزشکار که با ورزشکاران جور شده بودند انتخاب و طی یک مطالعه مقایسه‌ای مورد – شاهدی، حس وضعیت مفصل گلنوهومرال و حس حرکت آنها با استفاده از دستگاه سی.پی.ام مورد مقایسه قرار گرفت. از آزمون آماری تی‌مستقل برای تحلیل داده‌ها استفاده گردید. یافته‌ها: افزایش معناداری در دامنه چرخش خارجی والیبالیست‌ها (۰/۰۳۵=P) و کاهش معناداری در خطای باز سازی زاویه آنها (۰/۰۱۷=P) نسبت به غیرورزشکاران وجود داشت. در حالی‌که هیچ‌گونه تفاوت معناداری در آستانه درک حرکت بین دو گروه مشاهده نشد (۰/۳۹۲=P). نتیجه‌گیری: والیبالیست‌ها دارای دامنه چرخش خارجی بیشتر و دقت بازسازی زاویه بالاتری در اندام فوقانی غالب خود می‌باشند. شانه پرتاب‌کننده به طور مکرر در معرض حرکات چرخشی شدید می‌باشد. این منجر به تطابق عصبی عضلانی می‌شود و بدین ترتیب می‌تواند باعث بهبود حس‌ عمقی گردد.

Title: بررسی ارتباط وضعیت شناختی با میزان استقلال در فعالیت‌های روزمره زندگی در افراد راست‌دست مبتلا به سکته‌ مغزی

http://rehabilitationj.uswr.ac.ir/article-1-333-fa.html

هدف: ارزیابی میزان تأثیر آسیب‌های ناشی از سکته‌مغزی بر فعالیت‌های روزمره بیماران مبتلا، جهت برنامه‌ریزی اقدامات توانبخشی در ارتقای تأمین استقلال آنان شایان توجه است. لذا این پژوهش با هدف تعیین ارتباط آسیب عملکرد شناختی و میزان استقلال فردی در بیماران سکته‌مغزی انجام گرفته است.   روش بررسی: این پژوهش، یک مطالعه توصیفی- تحلیلی به شیوه مقطعی بود. 25 بیمار سکته‌مغزی بر اساس معیارهای پژوهش و از بین بیماران سه بیمارستان و یک مرکز تخصصی توانبخشی انتخاب و توسط شاخص فعالیت‌های روزمره بارتل (جهت تعیین استقلال در فعالیت‌های روزمره) و آزمون شناختی لوتکا (جهت بررسی وضعیت شناختی) مورد ارزیابی قرار گرفتند. نمونه‌گیری به روش ساده از جامعه دردسترس انجام شد. در نهایت، ارتباط وضعیت شناختی و میزان استقلال فردی از طریق آزمون همبستگی و رگرسیون خطی بررسی شد. یافته‌ها: یافته‌های آماری ارتباط میان نمره کل آزمون لوتکا و شاخص بارتل را رد کرد (P=0/087). در عین حال برخی از اجزای این دو آزمون با یکدیگر مرتبط بودند. نتیجه‌گیری: اگرچه عدم ارتباط دو آزمون لوتکا و بارتل تا حدی به دلیل پایین بودن شدت آسیب شناختی در نمونه‌های مورد مطالعه است، اما توجه به این نکته نیز لازم است که شاخص بارتل تنها براستقلال و عدم استقلال فردی تأکید داشته و به نحوه اجرای فعالیت‌ها نمی‌پردازد. از این رو لازم است جهت بررسی و ارزیابی فعالیت‌های روزمره از آزمون‌هایی استفاده شود که قابلیت تحلیل فعالیت‌ها را دارند.

Title: مقایسه فعالیت الکتریکی گروهی ازعضلات اطراف شانه در حین انجام ورزشهای زنجیره بسته در افراد جوان سالم

http://rehabilitationj.uswr.ac.ir/article-1-334-fa.html

هدف: در مورد اثر میزان استحکام سطح اتکا در حین تمرینات، بر ازدیاد فعالیت عضلات اطراف شانه تناقضات زیادی وجود دارد. هدف از انجام مطالعه حاضر بررسی ارتباط بین تغییرات میزان ثبات سطح اتکا با میزان فعالیت الکترومیوگرافیک استاتیک عضلات اطراف شانه است. روش‌ بررسی: در این تحقیق تجربی 30 فرد سالم مطابق معیارهای مورد نظر از نمونه‌های در دسترس انتخاب و فعالیت الکترومیوگرافیکی سطحی عضلات ذوزنقه‌ای‌ فوقانی، ذوزنقه‌ای‌ تحتانی، شانه‌ای‌قدامی، سر دراز دو سر، گرد بزرگ و سه‌گوش‌خلفی سمت غالب آنها در حالت استراحت و پس از 10 ثانیه قرار گرفتن در 6 وضعیت مختلف تست شنا رفتن، با توالی تصادفی و متفاوت از نظر میزان استحکام سطح اتکا، ثبت گردید. میزان مجذور ریشه میانگین سه ثانیه میانی الکترومیوگرافی برای مقایسه شدت فعالیت عضلانی بین وضعیت‌ها مورد استفاده قرار گرفت. سپس داده‌ها با آزمون‌های تحلیل‌ واریانس با اندازه‌گیری‌های مکرر و همبستگی پیرسون مورد بررسی قرار گرفتند. یافته‌ها: شدت فعالیت عضلانی در سه وضعیتی که سطح اتکای مستحکم استفاده شد، بیشتر از وضعیت‌هایی بودندکه ثبات سطح اتکا کمتر شده بود. این تفاوت قابل توجه در تمام عضلات غیر از عضله ذوزنقه‌ای‌فوقانی (۰/۰۸۱=P) معنادار بود (۰/۰۰۱>P). نتیجه‌گیری: ازدیاد بی‌ثباتی سطح اتکا نمی‌تواند به عنوان یک روش جهت ازدیاد پیشرونده قدرت عضلات اطراف شانه مورد استفاده قرار گیرد.

Title: مقایسه تأثیر ارتز بی‌حرکت‌کننده و گچ‌گیری بر راستای مفصل کف‌پایی-انگشتی در بیماران هالوکس‌والگوس پس از عمل جراحی

http://rehabilitationj.uswr.ac.ir/article-1-337-fa.html

هدف: این تحقیق با هدف مقایسه تأثیر ارتز بی‌حرکت‌کننده و گچ‌گیری بر راستای مفصل کف‌پایی - انگشتی در بیماران هالوکس‌والگوس پس از عمل جراحی صورت گرفت. روش بررسی: در این مطالعه شبه‌تجربی و مداخله‌ای 24 بیمار هالوکس‌والگوس واجد شرایط (سن 19 تا 50 سال، زاویه هالوکس‌والگوس حداکثر 25 درجه، زاویه بین استخوان‌های کف‌پایی اول و دوم 11 تا 13 درجه، عدم تغییرات دژنراتیو در مفصل و عدم وجود بیماری‌های سیستمیک)، از بین مراجعه‌کنندگان به درمانگاه ارتوپدی بیمارستان میلاد تهران به‌طور ساده و در دسترس انتخاب و براساس بلوک‌های تصادفی تبدیل شده، در دو گروه اسپلینت و گچ‌گیری قرار گرفتند. در گروه اسپلینت، قبل از عمل جراحی از پای بیمار قالب گرفته شد و بلافاصله بعد از عمل، اسپلینت بر روی پای بیمار بسته شد. در گروه گچ‌گیری پای بیمار تا حدود ۵ سانتی‌متر بالای مچ توسط پزشک متخصص گچ‌گیری شد. از کل بیماران بلافاصله و 6 هفته بعد از عمل رادیوگرافی به‌عمل آمد و زاویه هالوکس‌والگوس آنها مقایسه گردید. برای تحلیل داده‌ها از آزمون‌های آماری مجذور خی، تی‌مستقل، تی‌زوجی، تحلیل واریانس یکطرفه و تحلیل کوواریانس استفاده شد. یافته‌ها: هیچ‌گونه تفاوتی در اختلاف میانگین‌های زاویه هالوکس‌والگوس بلافاصله بعد از عمل و 6 هفته بعد از عمل دو گروه وجود نداشت و دو گروه از این نظر اختلاف معناداری نداشتند(۱/۰۰=P). مقایسه میانگین زاویه هالوکس‌والگوس بلافاصله و 6 هفته بعد از عمل جراحی در هر یک از گروه‌ها به تفکیک نیز اختلاف معناداری نداشت (۱/۰۰=P). نتیجه‌گیری: تأثیر گچ‌گیری و اسپلینت در حفظ زاویه هالوکس‌والگوس بعد از عمل جراحی اصلاح این دفورمیتی یکسان بوده و لذا می‌توان از هر دو روش به‌عنوان مراقبت‌های محافظتی به قصد حفظ موقعیت اصلاح شده به فراخور وضعیت بیمار استفاده نمود.

Title: تأثیر روان‌نمایشگری بر افزایش مهارت‌های اجتماعی و عزت‌نفس بیماران مبتلا به اسکیزوفرنیای نوع یک و دو

http://rehabilitationj.uswr.ac.ir/article-1-338-fa.html

هدف: اختلال در مهارت‌های اجتماعی ویژگی بارز بیماران اسکیزوفرنیاک است. هدف اصلی مطالعه حاضر بررسی تأثیر روان‌نمایشگری بر افزایش مهارت‌های اجتماعی و عزت‌نفس بیماران مبتلا به اختلال اسکیزوفرنیای نوع I و II می‌باشد. روش بررسی: در این مطالعه تجربی و مداخله‌ای از بین بیماران مرد مبتلا به اسکیزوفرنیا که در طول شش ماه اول سال 1384 در بخش‌های اعصاب و روان بیمارستان روانپزشکی ایثار شهر اردبیل بستری بودند، 80 نفر به روش نمونه‌گیری تصادفی ساده انتخاب شده و به‌صورت تصادفی‌تعادلی به چهار گروه 20 نفره تخصیص یافتند. جلسات اجرایی روان‌نمایشگری برای گروه‌های آزمایش نووع یک و دو به مدت ۹ هفته و هر هفته ۳ جلسه شامل مراحل آمادگی، اجرا و درون‌پردازی و تمرین رفتاری برای جمع‌آوری داده‌های پژوهش، از پرسشنامه اطلاعات جمعیت‌شناختی، فهرست مهارت‌های اجتماعی ویژه بیماران مبتلا به اسکیزوفرنیا و مقیاس عزت‌نفس روزنبرگ استفاده شد. داده‌ها با استفاده از آزمون تی‌مستقل و تحلیل واریانس دو عاملی تجزیه و تحلیل شد. یافته‌ها: تفاضل میانگین نمرات پیش‌آزمون- پس‌آزمون مهارت‌های اجتماعی (۰/۰۰۱>P) و عزت‌نفس (۰/۰۰۵=P) بین گروه آزمایش و کنترل (کل بیماران) تفاوت معنادار وجود داشت. بین گروه‌های آزمایش نوع ۱ و نوع ۲ از نظر میانگین نمرات پیش‌آزمون (۰/۰۰۳=P)، پس‌آزمون (۰/۰۰۱>P) و تفاضل پیش‌آزمون- پس‌آزمون (۰/۰۰۹=P) مهارت‌های اجتماعی و همچنین از نظر میانگین نمرات پیش‌آزمون (۰/۰۵=P)، پس‌آزمون (۰/۰۰۱>P) و تفاضل پیش‌آزمون- پس‌آزمون (۰/۰۳۵=P) عزت‌نفس تفاوت معناداری وجود داشت. بین گروه‌های آزمایش و کنترل نوع یک اسکیزوفرنیا از نظر تفاضل نمرات پیش‌آزمون و پس‌آزمون مهارت‌های اجتماعی (۰/۰۰۱>P) و عزت‌نفس (۰/۰۰۱>P) و همچنین گروه‌های آزمایش و کنترل نوع دو اسکیزوفرنیا در تفاضل نمرات پیش‌آزمون- پس‌آزمون مهارت‌های اجتماعی (۰/۰۱=P) و عزت‌نفس (۰/۰۳=P) تفاوت معنادار وجود داشت. نتیجه‌گیری: اجرای فنون روان‌نمایشگری برمهارت‌های اجتماعی و عزت‌نفس بیماران مبتلا به اسکیزوفرنیا مؤثر است و این اثر بر بیماران مبتلا به نوع یک این اختلال بیشتر است.
